# Symposium ‘understanding and managing satiety: processes and opportunities’

**DOI:** 10.1017/jns.2020.32

**Published:** 2020-09-03

**Authors:** Giuseppina Mandalari

**Affiliations:** Department of Chemical, Biological, Pharmaceutical and Environmental Science, University of Messina, Vill. SS, Annunziata, 98168 Messina, Italy

**Keywords:** almonds, satiety, food structure

## Abstract

This brief report summarises a framework for understanding satiety presented at the 13th European Nutrition Conference, FENS 2019 – Malnutrition in an Obese World: European Perspectives. Aspects of satiety phenotyping and role of food hedonics in satiation are considered in the context of appetite control and obesity. Almonds are evaluated for their unique composition and structure which affect their behaviour in the human gastrointestinal tract. Their role in appetite control and management of satiety has been explored.

## Introduction

The increase in worldwide prevalence of obesity has been significant over the last few decades, with approximately 30 % of obesity rate reported in the UK in 2019^(1^^)^. The challenges in managing appetite control are related to the increased prevalence of obesity. The biological drive to eat in human subjects, a component of appetite control, is a response to energy requirements. Fat-free mass and resting metabolic rate are major determinants of the drive to eat and are associated with the tonic drive^(2)^. This tonic drive to eat can be contrasted by other aspects of appetite influenced by culture and environment. These include types of foods available, food choices, appropriateness perceptions, where and how to eat.

Almonds (*Prunus dulcis* L.) are a high-nutrient and caloric-density food with beneficial properties in terms of appetite control. Their effect could be due to the high protein and fibre content, low digestible energy associated with slow energy release, post-ingestive satiety, reduced wanting to eat high-fat foods and enhanced satiety responsiveness^(3–6)^. A recent study demonstrated that raw almonds effectively controlled appetite compared to an energy matched alternative snack^(7)^.

The present report highlights some findings presented at the 13th European Nutrition Conference, FENS 2019 – Malnutrition in an Obese World: European Perspectives. A framework for understanding satiety and the role played by food hedonics in appetite control and satiety is summarised. Almonds are considered for their unique structure and their role in satiety.

## Symposium presentations

The symposium ‘Understanding and managing satiety: processes and opportunities’, funded by the Almond Board of California, was organised into four presentations, as follows:

1. Interactions among the Drive to Eat, Satiation and Satiety – with Relevance for Obesity (Prof. John E. Blundell, University of Leeds, Leeds, UK).
2. The Structure of Plant-based Food and Nutrients Bioaccessibility – Implications for Satiety and Metabolisable Energy (Dr Myriam Grundy, University of Reading, Reading, UK).
3. Towards an Understanding of Almond Digestion and Its Effect on Satiety: Cell Walls, Processing and Particle Size (Dr Giuseppina Mandalari, University of Messina, Messina, Italy).
4. The Role of Food Hedonics – Liking and Wanting – in Satiation and Satiety: Opportunities for Functional Foods (Prof. Graham Finlayson, University of Leeds, Leeds, UK).

**Prof. Blundell** started the symposium by giving an overview of obesity and a framework for understanding satiety. People with obesity are not trying to get fat; obesity just seems to happen. Equally, most people are not trying to eat beyond their caloric needs; overconsumption just seems to happen. Inhibitory processes do not seem to be strong enough to prevent overconsumption: could they be strengthened?

The human appetite system is intimately linked to body composition and therefore to obesity. Appetite, by definition, influences energy intake (food consumption) and the associated motivational states, such as hunger. Appetite is also influenced by energy expenditure (metabolic and behavioural). Three aspects should be considered: the origins of the drive to eat (hunger), food choice and the control over the inhibition of eating, and the amount of food consumed. Obesity is an emergent property of a complex system; it may arise from a strong drive to eat, inappropriate food choices or weak inhibition of eating.

Satiety is an important psycho-biological process involved in the expression of human appetite, which inhibits hunger and intake following consumption of a food or beverage^(8)^. It is part of a system of human appetite control: overconsumption arises from an imbalance between the tonic biological drive to eat (metabolic drive), and inhibitory tonic and episodic processes.

**Fig. 1.**
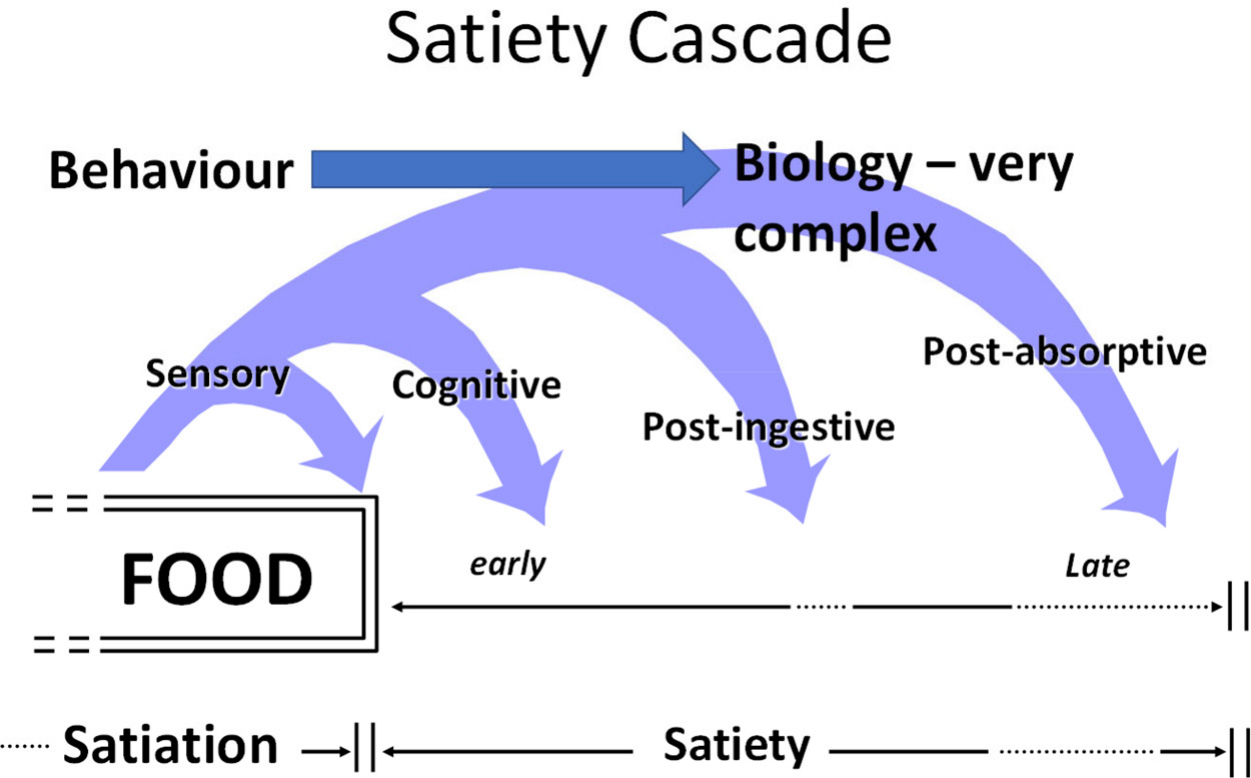
Satiety cascade^(7)^.

Satiety can be viewed as an emergent property of complex GI physiology, arising through the integration of numerous factors, including cognitive, sensory, post-ingestive and post-absorptive signals, all conceptualised in the Satiety Cascade (Fig. 1)^(7)^.

Although all foods can induce satiety, the intensity and duration of the inhibition can vary through two major processes:

- the physiology of the gastrointestinal tract (GIT) and
- any sensory and cognitive features of the foods chosen and ingested.

Appetite-related peptides, such as ghrelin, cholecystokinin (CCK), glucagon-like peptide 1 (GLP-1) and peptide YY (PYY), are thought to play a role in the episodic control of appetite and are known to fluctuate around mealtimes. These peptides are released from several sites throughout the GIT: ghrelin is released from the stomach and is often referred to as the ‘hunger hormone’, whereas CCK, GLP-1 and PYY are released from the small and large intestines and are considered satiety peptides^(9)^.

Further research has demonstrated that the nature of the foods chosen influences both the strength of satiation (amount eaten) and satiety (after eating)^(10)^. Foods can help consumers control their appetite, eat healthily and manage their weight through a macronutrient satiating power derived by their high proportion of protein and fibre (low-energy density).

Therefore, there is potential for foods to influence GI physiology in different ways and achieve distinct effects on satiety.

All foods induce satiety to a variable strength, but satiety responsiveness is highly variable from person to person. This led to the identification of high and low satiety phenotypes^(11)^, with marked differences in major markers of satiety following either a low-energy density or a high-energy density meal. People with low satiety responsiveness show a more rapid rise in hunger after a meal and are at risk of overconsumption^(12)^.

Many opportunities can be explored to manipulate foods in order to optimise satiety, and specific natural foods can be further explored for their important satiety-inducing properties.

During her presentation, **Dr Grundy** highlighted the importance of considering the overall structure of the food and not only its composition. In order to promote and/or develop food products with targeted health benefits, it is essential to understand the mechanisms by which macronutrients are released and digested in the GIT. Nutrients’ behaviour in the GIT affects physiological responses, including satiety. Since food structure has an effect on nutrient delivery and metabolisable energy, it is possible to control the bioaccessibility of nutrients and satiety. With the term ‘bioaccessibility’, we refer to the proportion of a nutrient or phytochemical compound ‘released’ from a complex food matrix during digestion and, therefore, potentially available for absorption in the GIT.

It is well accepted that lipid bioaccessibility and digestibility have implications on risk factors of cardiometabolic diseases and plant food structure. In particular, dietary fibre has an impact on lipid release and digestibility *via* the following mechanisms:

- encapsulation of lipids and other phytochemicals;
- increase in viscosity of intestinal contents;
- interaction with agents of digestion (e.g. bile salts and lipases);
- interaction with the mucus layers.

Despite their high lipid content, almond consumption is associated with reduced risk of cardiovascular disease^(13,14)^. One explanation for this paradox could be the limited bioaccessibility of almond lipids: the cell wall matrix represents a physical barrier in the upper GIT. When measuring the rate and extent of lipolysis of raw and roasted almonds using an *in vitro* duodenum digestion model, particle size had a crucial effect. In particular, a decrease in particle size led to an increased rate and extent of lipolysis and separated cells with intact cell walls showed the lowest levels of digestibility^(15)^.

Mastication studies using natural and roasted almonds indicated that most of the lipid in the bolus is not immediately bioaccessible and therefore remains unavailable for early stages of digestion. Microstructural examination showed that most intracellular lipid remained unmodified in intact cells after mastication^(16)^. The role of almond cell wall encapsulation and porosity in regulating lipolysis has also been demonstrated in digestibility experiments using raw, roasted almonds and isolated almond oil bodies: significant differences in the rates and in the extent of lipolysis were observed between almond cells and oil bodies, regardless of the enzymes used^(17)^.

The lipid encapsulation mechanism could provide an explanation for why almonds have a low metabolisable energy content and an attenuated impact on post-prandial lipemia. The slow release of lipids into the small intestine causing GLP-secretion could result in a greater satiating potential and a reduced appetite related to almond consumption^(18)^. The greater suppression of hunger observed with almond snacking compared to crackers, associated with increased concentrations of post-prandial glucagon-like peptide-1 and reduced decline in insulin, could also be mediated by differences in oral mechanical effort (greater chewing reported with almonds)^(19)^.

Another food to investigate is oat, rich in phytochemicals with various properties, all likely to influence human lipid metabolism. Since oat β-glucan plays a positive role in influencing lipid and cholesterol metabolism, the release of β-glucan, and the rate and extent of lipolysis were measured in the presence of different sources of oat β-glucan during digestion. The findings showed that the positive action of β-glucan was likely to involve complex processes and interactions within the food matrix used^(20)^. In fact, free fatty acid release was affected more by complex oat materials than purified forms of β-glucan. The simulated digestion process appeared to have little or no effect on the β-glucan solubilisation in any of the oat samples.

Amaranth is another example of plant food whose structure and processing have an effect on functionality. It is particularly rich in protein, dietary fibres and micronutrients and represents a staple food in certain countries, such as Kenya. The digestibility of amaranth lipid during simulated digestion appeared higher in raw amaranth flour compared with other products, such as raw grain, toasted grain and flour.

Taken all together, these data demonstrated that the rate and extent of lipolysis were reduced when the degree of complexity of the food material increased, though the processes that impact on lipolysis differ according to the plant studied. Slow energy release in the gut, correlated to a significant proportion of lipid encapsulation, is likely to be beneficial in prolonging a feeling of fullness and regulate appetite control.

**Dr Mandalari**'s presentation focused on the effect of food matrix and processing on the bioaccessibility of almond nutrients from almond seeds during digestion using bio-relevant *in vitro* models which allow a detailed investigation of the behaviour of foods in the gut prior to perform *in vivo* studies. A Dynamic Gastric Model (DGM) was used to simulate the human stomach, both from a biochemical point of view through the addition of acid and enzyme secretions and from a physical processing mechanism, to simulate the contractions of the antrum. A controlled delivery to the small intestine is achieved by a preferential sieving mechanism of the food particles. The gastric digesta samples were then incubated under duodenal conditions with the addition of pancreatic enzymes, bile salts and surfactants.

The concept of food structure and health is a multidisciplinary approach to understand the relationship between the structure of foods and a physiological response. A number of factors affect bioaccessibility, including food structure and preparation, processing, as well as the digestive system (upper and lower GIT).

Almond cell walls are rich in non-starch polysaccharides, particularly arabinose-rich polysaccharides, with a high concentration of phenolic compounds. During disruption of almond tissue either by mechanical methods or through chewing, only the first layer of cells at the fractured surface is ruptured and therefore able to release lipid^(21)^. Between 7⋅8 and 11⋅1 % of the total lipid was released from almonds as a result of mastication, with no significant differences between natural and roasted almonds. An increase in the percentage of lipid release was detected after *in vitro* gastric (16⋅4 and 15⋅9 %) and duodenal digestion (32⋅2 and 32⋅7 %) for natural and roasted almonds, respectively^(22)^. The composition of almond cell walls was not affected by processing or simulated digestion. These findings confirmed that almond cell walls prevent lipid release from intact cells, providing a mechanism for incomplete nutrient absorption in the gut.

**Fig. 2.**
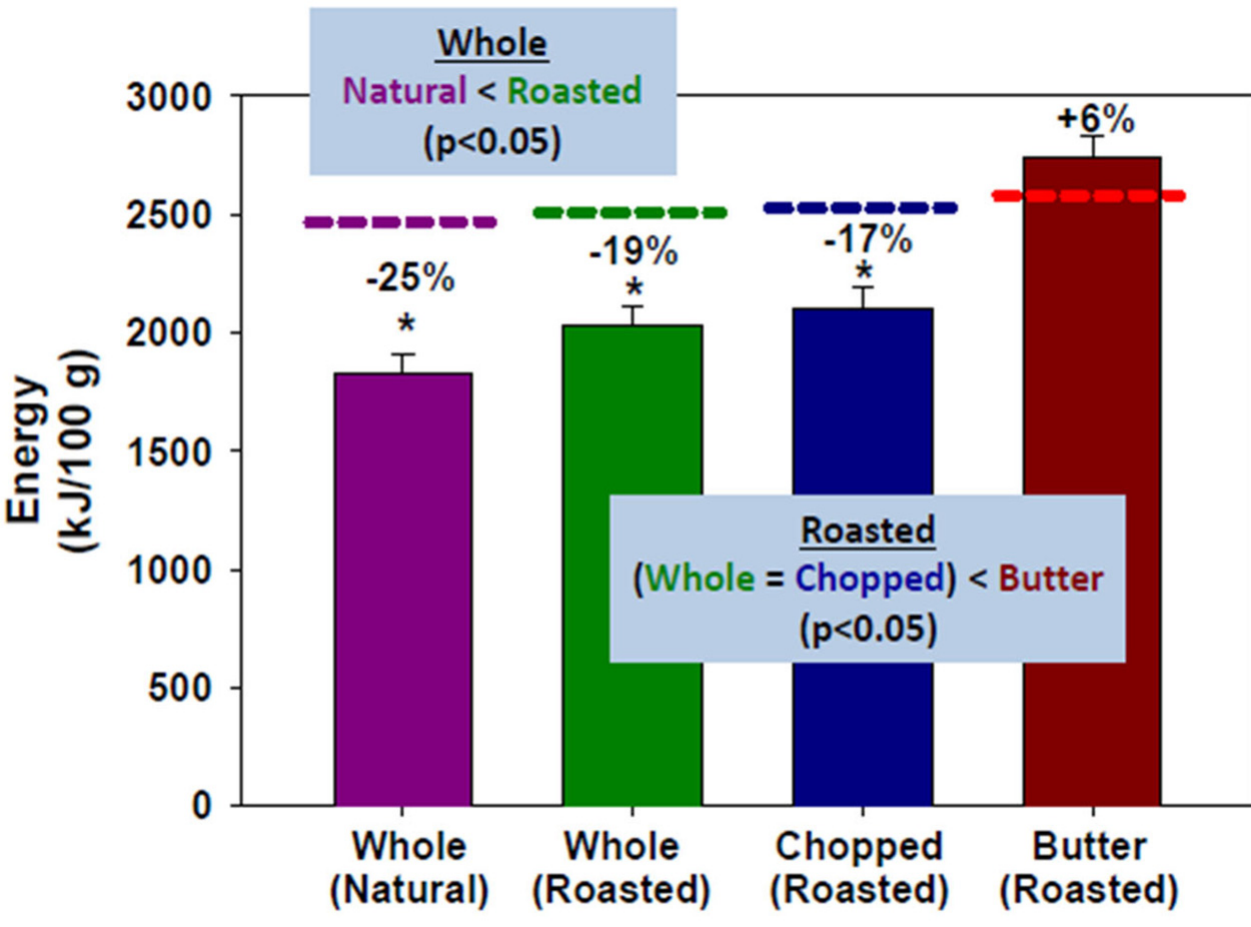
Metabolisable energy loss compared to that calculated from nutrient composition using the Atwater general factors for whole natural almonds (purple), roasted almonds (green), chopped roasted almonds (blue) and roasted almond butter (red)^(24)^.

When an almond-rich diet was fed to healthy volunteers, intact cotyledonary cells, in which the cell walls encapsulated intracellular lipid, were detected in the faecal samples and the lipid appeared susceptible to bacterial fermentation. However, the structure and particle size of the almond meals are the most important factors in regulating lipid bioaccessibility in the gut: Microstructural analysis of faecal samples from volunteers consuming natural almonds, roasted almonds, diced almonds and almond butter confirmed that some lipid in natural, roasted and diced almonds was retained and remained encapsulated within the plant tissue throughout digestion, whereas almost complete digestion was observed in the almond butter sample^(23)^. Particle size had a crucial impact on lipid bioaccessibility since it is an indicator of the proportion of ruptured cells in the almond tissue. Separated almond cells with intact cell walls showed the lowest levels of lipid digestibility. These findings provided a mechanistic understanding of the loss in metabolisable energy compared to that calculated from nutrient composition using the Atwater general factors (Fig. 2)^(24)^.

Given that lipid bioaccessibility is highly dependent on particle size and cell diameter, a mathematical model was developed to predict lipid release from almonds in the upper GIT^(25)^. Further work is warranted to evaluate the efficacy of this model to accurately predict nutrient bioaccessibility in a broad range of edible plants.

In conclusion, the data presented showed that the presence of the food matrix affected the rate and extent of nutrient release in the gut and the low digestibility of lipid from almonds were strictly related to the structure of the meal and the particle size distribution. Therefore, food structure has the potential to influence satiety through nutrient bioaccessibility in the gut.

**Prof. Finlayson** introduced the concept of food pleasure as a primary determinant of food choice, thus affecting appetite and food intake. From a homeostatic perspective, the drive and inhibition of food intake are a net balance of signals influencing how much food is consumed. Consumers need to be motivated and rewarded for meeting their biological nutrient requirements, but they are also responsive to environmental variances in the food repertoire^(26)^. On the other hand, the hedonic drive from liking and wanting food helps determine what is eaten and when. The hedonic value of food is one of the most important attributes for consumers but also problematic for overeating and weight management. The distinction between liking and wanting may also have implications for identifying foods that are beneficial for appetite control.

The idea that snacking contributes to weight gain and obesity is also very popular among consumers. Various definitions of what constitutes snacking can be found in the scientific literature^(27)^. These include:

- eating frequency;
- small portions of packaged foods;
- sweets and desserts;
- unstructured eating throughout the day separate from standard meals.

A number of studies have directly examined the effect of snacking on body weight: no differences were detected between snacking and non-snacking on weight outcomes^(27)^. However, in any society, it is possible to distinguish between susceptible and resistant phenotypes, based on the fact that some individuals gain fat more easily than others^(28)^. The investigation of these phenotypes can help understand some of the causes leading to obesity. It is also possible to identify reliable susceptible phenotypes for appetite control, who may express weak satiation, fragile, satiety, obesogenic food choices, excessive wanting and liking. The low satiety phenotype proposed earlier by Prof. Blundell shows weaker suppression of hunger throughout the day and this phenotype consistently consumes more energy compared to the high satiety phenotype^(12)^.

Replacing unhealthy (high fat, high sugar and energy dense) snacks with more nutrient-dense foods could have a positive impact on diet quality and consequently on body weight changes. Individualised feeding strategies in low satiety phenotypes could affect the predisposition to snacking and the overall metabolic state.

The effect of meal-induced satiation on implicit and explicit processes of liking (L) and wanting (W) was evaluated by developing a computer-based procedure to measure L and W in hungry and satiated states^(29)^. The findings from this study provide support that implicit and explicit processes of food reward can be simultaneously measured and dissociated using a test meal. Using this approach, the low satiety phenotype had more liking (*P* < 0⋅05) and wanting (*P* < 0⋅01) for high-fat foods compared to the high satiety phenotype and was characterised by:

- higher levels of hunger across the day;
- greater energy intake;
- greater liking and wanting for high-fat foods;
- blunted post-prandial glucose and cortisol response, higher trait anxiety, shorter sleep duration and night eating symptoms.

The low satiety phenotype with obesity also showed poorer weight loss outcomes to structured weight management programs, a greater liking for high-fat foods after high-energy density meals and greater tendency to snack between high-energy density meals^(29)^.

A recent study compared the effect of consuming almonds as a mid-morning snack compared to an energy and weight-matched comparator snack (savoury crackers) or the equivalent weight of water (zero energy control). Results showed that almonds suppressed hedonic preference (implicit wanting) for consuming high-fat foods and demonstrated a higher satiety quotient than crackers. Importantly, almonds were perceived to have a more favourable consumer profile aligned with successful weight management^(30)^.

Almonds are rich in protein, fibre and low digestible fat, but relatively low in available carbohydrate. The role played by proteins and fibres on appetite control has been well-established^(31,32)^. With almonds, their effects may be cumulative due to the different mechanisms of action. The unique composition and structural features of almonds can have a potential impact on hunger and satiety control, and subsequent energy intake.

## Conclusions

The symposium ‘Understanding and managing satiety: processes and opportunities’ has demonstrated that satiety is an emergent property of a complex gastrointestinal physiology and its responsiveness varies greatly among individuals. Food-based approaches may help normalise both homeostatic and hedonic appetite responses in susceptible individuals. Highly satiating meals or snack foods (e.g. high fibre and high protein) can counteract loss of appetite control and limit energy intake.

The low digestibility of lipid from almonds is strictly related to the structure of the meal and the particle size distribution, with a significant proportion of lipid remaining encapsulated after *in vitro* and *in vivo* gastrointestinal digestion. The slow release of energy from almonds is likely to be beneficial in prolonging a feeling of fullness. Although the processes that impact on lipolysis differ according to the plant studied, it is believed that food structure and degree of processing are crucial for appetite control. Almonds are beneficial for appetite control regulation and management of satiety. Further studies on the slow energy release from almonds and other high-density foods are required to assess their longer-term effect on weight gain and satiety.
